# Supplementary material for: A whole-animal phenotypic drug screen identifies suppressors of atherogenic lipoproteins
Source: eLife. 2026 Aug 27;14:RP105314. doi: 10.7554/eLife.105314 (PMC13521566; doi:10.7554/eLife.105314)
Supplement: Supplementary file 2 [file elife-105314-code1.zip › JHDL_primary_result/Cinnamon oil (Cinnamon leaf oil)/Cinnamon oil (Cinnamon leaf oil)_JHDL_29_027_057_2019_12_06_13_27.pdf]

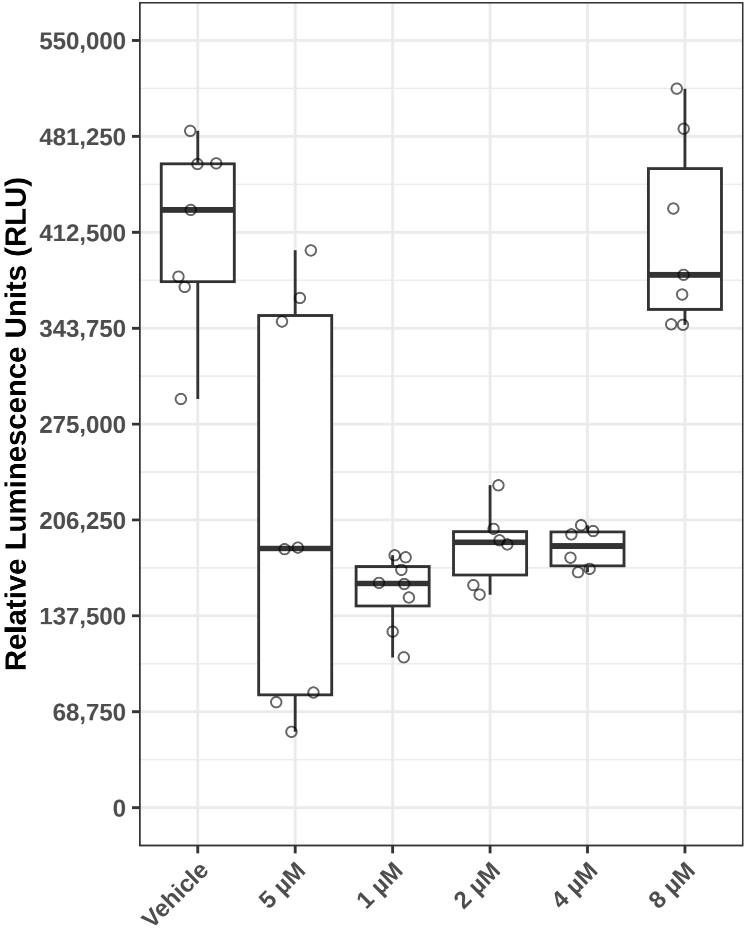
Relative Luminescence Units (RLU)
550,000
481,250
412,500
343,750
275,000
206,250
137,500
68,750
0
Vehicle
5 μM
1 μM
2 μM
4 μM
8 μM
